# Association of Borderline Personality Disorder Criteria With Suicide Attempts Among US Adults

**DOI:** 10.1001/jamanetworkopen.2021.9389

**Published:** 2021-05-11

**Authors:** Carlos M. Grilo, Tomoko Udo

**Affiliations:** 1 Department of Psychiatry, Yale University School of Medicine, New Haven, Connecticut; 2 Department of Health Policy, Management, and Behavior, School of Public Health, University at Albany, State University of New York, Rensselaer

## Abstract

**Importance:**

Rates of suicide are increasing. Although borderline personality disorder (BPD) and other psychiatric disorders are associated with suicide, there is a dearth of epidemiological research on associations between BPD and suicide attempts (SAs). Delineating the SA risk associated with BPD and its specific criteria in a nationally representative sample of individuals could inform recognition and intervention efforts for SAs.

**Objective:**

To examine the association of a BPD diagnosis and specific BPD criteria with SAs in US adults.

**Design, Setting, and Participants:**

This cross-sectional study analyzed data from the National Epidemiological Survey on Alcohol and Related Conditions–III (NESARC-III), a psychiatric epidemiological survey of noninstitutionalized US adults aged 18 or older conducted from April 2012 to June 2013. Eligible adults were randomly selected from households within census-defined counties or groups of counties. Data were analyzed from December 2020 to January 2021.

**Main Outcomes and Measures:**

Prevalence of *Diagnostic and Statistical Manual of Mental Disorders* (Fifth Edition) psychiatric and personality disorders, BPD and its specific criteria, SAs, and adverse childhood experiences (ACEs), as assessed by structured diagnostic or clinical interviews; prevalence is expressed as weighted means. Multivariable-adjusted logistic regression was used to compare the risk of lifetime and past-year SAs by BPD diagnosis and by each specific BPD criterion. Analyses were adjusted for demographic and clinical factors, including psychiatric comorbidity, age at BPD onset, and ACEs.

**Results:**

Of 36 309 respondents, 20 442 (56.3%) were women and 52.9% were non-Hispanic White; the mean (SD) age was 45.6 (17.5) years. The prevalence (SE) of lifetime and past-year SAs among participants with a lifetime diagnosis of BPD based on original NESARC-III diagnostic codes was 22.7% (0.8%) (adjusted odds ratio [AOR], 8.40; 95% CI, 7.53-9.37) and 2.1% (0.2%) (AOR, 11.77; 95% CI, 7.86-17.62), respectively. With use of diagnostic codes requiring 5 BPD criteria to meet social-occupational dysfunction, prevalence (SE) of lifetime and past-year SAs was 30.4% (1.1%) (AOR, 9.15; 95% CI, 7.99-10.47) and 3.2% (0.4%) (AOR, 11.42; 95% CI, 7.71-16.91), respectively. After excluding the BPD criterion of self-injurious behavior (to eliminate criterion overlap), the prevalence (SE) of lifetime and past-year SAs was 28.1% (1.1%) (AOR, 7.61; 95% CI, 6.67-8.69) and 3.0% (0.4%) (AOR, 9.83; 95% CI, 6.63-14.55), respectively. In analyses adjusting for sociodemographic variables, psychiatric disorders, age at BPD onset, and ACEs, BPD diagnosis and specific BPD criteria of self-injurious behaviors and chronic feelings of emptiness were significantly associated with increased odds of lifetime SAs (BPD diagnosis: AOR, 2.10; 95% CI, 1.79-2.45; self-injurious behaviors: AOR, 24.28; 95 CI, 16.83-32.03; feelings of emptiness: AOR, 1.58; 95% CI, 1.16-2.14) and past-year SAs (BPD diagnosis: AOR, 11.42; 95% CI, 7.71-16.91; self-injurious behaviors: AOR, 19.32; 95% CI, 5.22-71.58; feelings of emptiness: AOR, 1.99; 95% CI, 1.08-3.66). In analysis with BPD criteria simultaneously entered (excluding self-injurious behavior), chronic feelings of emptiness were significantly associated with increased odds of lifetime SAs (AOR, 1.66; 95% CI, 1.23-2.24) and past-year SAs (AOR, 2.45; 95% CI, 1.18-5.08).

**Conclusions and Relevance:**

In a national sample of adults, after adjusting for demographic and clinical variables, a BPD diagnosis and the specific BPD criteria of self-injurious behaviors and chronic emptiness were significantly associated with increased SA risk. Although BPD is a complex heterogeneous diagnosis, the results of this study suggest that the criteria of self-injurious behaviors and chronic feelings of emptiness should be routinely considered in suicide risk assessment.

## Key Points

**Question:**

Are borderline personality disorder (BPD) and its specific criteria associated with suicide attempts among US adults?

**Findings:**

In this cross-sectional study of a nationally representative sample of 36 309 US adults, after adjusting for demographic and other clinical variables, lifetime BPD diagnosis and the specific criteria of self-injurious behaviors and chronic feelings of emptiness were significantly associated with increased risk for lifetime and past-year suicide attempts.

**Meaning:**

The findings suggest that specific BPD criteria of self-injurious behaviors and chronic feelings of emptiness should be routinely considered in suicide risk assessment.

## Teaser

This cross-sectional study uses data from the National Epidemiologic Survey on Alcohol and Related Conditions to examine associations between specific criteria of borderline personality disorder and suicide attempts in US adults.

## Introduction

Suicide is a complex major public health problem nationally and globally.^1^ It represents the 10th leading cause of mortality in the US,^2^ and the rates of suicide and suicide attempts have increased substantially during the past 20 years.^3,4^ Research has identified some variables associated with risk for suicidal behaviors.^5^ In terms of dynamic and potentially malleable factors, psychiatric disorders are associated with suicide attempts (SAs)^4^ and suicide.^6^

Borderline personality disorder (BPD), which includes frequent self-injurious behaviors as 1 possible diagnostic criterion, is associated with substantial health care use^7^ and with SAs.^8,9,10^ Borderline personality disorder is also associated with high rates of psychiatric comorbidity^11^; however, studies^10,12,13^ using a clinical sample of treatment-seeking individuals found that the association observed between BPD and prospective risk for suicide behaviors occurred independently of psychiatric comorbidities. Using data from a 10-year longitudinal study with treatment-seeking patients with personality disorders or major depressive disorder without personality disorders, Yen and colleagues^13^ reported that among multiple psychiatric disorders, BPD was the most robust factor associated with prospectively observed SAs, and the association persisted even after adjusting for demographic (sex, educational level, and employment) and clinical (childhood sexual abuse, substance use disorders, and posttraumatic stress disorder [PTSD]) variables.

Yen and colleagues^12,13^ also found that certain specific criteria of the BPD diagnosis were significantly associated with SAs. This finding is important for multiple reasons. First, BPD is a complex heterogeneous diagnostic construct.^7^ Because the *Diagnostic and Statistical Manual of Mental Disorders* (Fifth Edition) (*DSM-5*)^14^ requires that any 5 of the 9 possible criteria for BPD be met, there are at least 126 possible combinations of criteria that could indicate a minimal threshold for the diagnosis. In addition, the 9 criteria (unstable relationships, affective instability, abandonment fears, anger, identity disturbance, chronic emptiness, dissociation, impulsivity, and self-injurious behavior) are heterogeneous, comprising traits, behaviors, and symptoms.^7,15^ One of the BPD criteria involves repetitive self-injurious behavior, which both can reflect SAs and is associated with SAs,^9,16^ and this creates the problem of criterion overlap when trying to assess risk for SAs. These complexities notwithstanding, delineating specific features of BPD that are associated with increased risk for SAs could clinically inform early identification and targeted intervention efforts. Research with diverse clinical samples has produced isolated findings suggesting associations between SAs and some of these variables, including affective instability,^9,12^ dissociation,^9^ impulsivity,^17^ and chronic emptiness.^18^ Yen and colleagues,^13^ in their analysis of all BPD criteria (adjusting for sociodemographic and related clinical variables), found that the specific criteria of identity disturbance, chronic feelings of emptiness, and frantic efforts to avoid abandonment were significantly associated with SAs.

Various potential confounds may limit the representativeness of findings from treatment-seeking and clinical studies. Persons who seek treatment for a concern and who also participate in research may differ from persons who do not seek treatment or choose not to participate in research at the treatment facility. Complex factors characterize treatment seeking,^19^ and various disparities (eg, racial/ethnic, sex, and economic) exist for different types of facilities for all psychiatric disorders.^20,21^ Representativeness of clinical samples has also long been questioned^22^ because of the well-known mathematical bias (ie, Berkson bias) that a person with 2 or more disorders can seek treatment for either disorder as well as the related, yet distinct, clinical biases whereby different comorbidities are associated with whether treatments are sought, and if so, for which disorder.^21,22^ Given high rates of comorbidity among patients with BPD,^11,23^ studies with clinical samples may be especially confounded and skewed by more severe clinical presentations.

The National Epidemiologic Survey on Alcohol and Related Conditions (NESARC) was a large epidemiological study with a representative sample of noninstitutionalized US adults that aimed to assess *DSM-5-*defined psychiatric disorders.^24,25^ The third wave of the study (NESARC-III; data collection in 2012-2013) was, to our knowledge, the first large psychiatric epidemiological study of US adults since the *DSM-5* was published. Earlier NESARC studies (waves I-II) served as an important first step toward filling a major gap in the research field of personality disorders that primarily relied on clinical treatment-seeking samples despite longstanding calls for studies using epidemiological data.^26^ One report based on the NESARC-III found a significantly elevated risk of meeting a diagnosis of BPD among participants who reported a recent SA^4^; however, neither the NESARC-III nor other epidemiological work, to our knowledge, has examined the rate of SAs in persons with BPD and considered associations with specific BPD criteria.

The present study aimed to examine whether a lifetime BPD diagnosis and specific criteria of BPD are associated with lifetime and past-year SAs in US adults after adjusting for other known sociodemographic and clinical variables associated with SAs, including childhood adverse experiences and psychiatric comorbidity. This study applied a more rigorous BPD diagnostic scoring method than was used in all 3 NESARC waves given substantial clinical and empirical concerns.^27^ This first analysis with a nationally representative sample examined some of the potential confounds attributable to treatment-seeking clinical biases in the study by Yen and colleagues.^13^ We hypothesized that BPD and the specific criterion of self-injurious behaviors would be significantly associated with elevated SA risk even after adjusting for relevant sociodemographic and clinical variables, including psychiatric and personality disorder comorbidity. We also sought to test whether the findings of Yen and colleagues^13^ from a clinical sample regarding independent associations between SA risk and 3 additional specific criteria (identity disturbance, chronic feelings of emptiness, and frantic efforts to avoid abandonment) would be found in a national sample of US adults.

## Methods

### Data Source and Study Population

This cross-sectional study used data from the NESARC-III, which aimed to estimate the prevalence of alcohol use and related conditions in noninstitutionalized US civilians aged 18 years or older.^24,25^ In the NESARC-III, multistage probability sampling was used with counties or groups of contiguous counties as primary sampling units, groups of census-defined blocks as secondary sampling units, and households within secondary sampling units as tertiary sampling units.^25^ Eligible adults were randomly selected from each household, but Hispanic, Black, and Asian household members were oversampled (ie, 2 respondents were selected from households with more than 4 eligible members who were racial/ethnic minorities). Of 59 725 households invited to participate, 42 692 (71%) completed the screening, and of the 43 364 eligible adults selected, 36 309 (84%) participated in the survey, for an overall response rate of 60.1%. Participants completed computer-assisted, face-to-face personal interviews conducted from April 2012 to June 2013 by 970 trained lay assessors with an average of 5 years of experience conducting health-related surveys. The NESARC-III received approval from the National Institutes of Health institutional review board, and participants provided oral informed consent.^25^ The present study was determined to be exempt from institutional review board approval and the need for informed consent by the institutional review board of the University at Albany, State University of New York, because it was an analysis of data from a previously approved survey. This study followed the Strengthening the Reporting of Observational Studies in Epidemiology (STROBE) reporting guideline.

### Diagnostic Assessment of Psychiatric Disorders

The NESARC-III used questions based on the National Institute on Alcohol Abuse and Alcoholism’s Alcohol Use Disorder and Associated Disabilities Interview Schedule-5 (AUDADIS-5),^28^ a structured interview that assesses a range of *DSM-5*–defined psychiatric disorders and their criteria, including lifetime BPD. The AUDADIS-5 also generates lifetime diagnoses for mood disorders (major depressive episodes, persistent depression, and bipolar 1 disorder), anxiety disorders (specific phobia, social phobia, panic disorder, agoraphobia, and generalized anxiety disorder), PTSD, substance use disorders (alcohol use disorder, drug use disorder, and nicotine use disorder), antisocial personality disorder, schizotypal disorder, and conduct disorder. Concordance between the AUDADIS-5 and the clinician-administered Psychiatric Research Interview for Substance and Mental Disorders, *DSM-5* version was fair to moderate for diagnoses (*k*, 0.24-0.72) and fair to excellent for dimensional measures of substance use disorders, mood disorders, anxiety disorders, and PTSD (intraclass correlation, 0.43-0.72).^29,30^ Test-retest reliability of the AUDADIS-5 for specific disorders was fair to excellent (*k*, 0.35-0.87) for diagnosis of substance use disorders, mood disorders, anxiety disorders, PTSD, and personality disorders.^31^

### Creation of Borderline Personality Disorder Criteria and Diagnosis

For a diagnosis of BPD, the *DSM-5* requires endorsement of any 5 of the 9 possible criteria and social-occupational dysfunction.^14^ A summary of how each BPD criterion was operationalized in this study using the specific AUDADIS-5 questions is provided in eTable 1 in the Supplement. The NESARC-III provided 2 BPD diagnostic codes, 1 requiring reported social-occupational dysfunction for at least 1 criterion and a second requiring reported social-occupational dysfunction for at least 2 criteria. This approach has been previously questioned as being too liberal because a report of marked distress or impairment in social function is a key characteristic of personality disorders.^27^ Thus, for this study, we created more stringent coding of each BPD criterion and the scoring of the BPD diagnosis. Specifically, we required that associated social-occupational dysfunction be reported for any specific criterion endorsed by the respondents; thus, to receive a BPD diagnosis, at least 5 criteria with associated dysfunction must have been endorsed. The NESARC-III also assessed the age when some of the BPD features began to occur; we used the same approximate time frame as the age at onset of BPD variables in our data analysis.

### Suicide Attempts

As part of their interview, NESARC-III respondents answered whether they had ever attempted suicide, and a total number of SAs in their lifetime was recorded for those who endorsed a history of SAs. In the NESARC-III data set, the question about the number of SAs was skipped (ie, recorded as missing) for those who reported no SA history; we coded this as 0 for our analysis of the number of SAs. Respondents were considered to have attempted suicide in the past year when the difference between their current age and the age at the time of the most recent SA was 0 or 1.

### Adverse Childhood Experiences

The NESARC-III included questions about 5 types of childhood maltreatment (physical neglect, emotional neglect, physical abuse, emotional abuse, and sexual abuse) by parents or caregivers and 13 other adverse events occurring before 18 years of age. Following previous studies,^32,33,34^ reporting of any form of childhood maltreatment or other adverse events was coded as having a positive history.

### Sociodemographic Characteristics

Respondents to the NESARC-III provided sociodemographic information, including age, sex, ethnicity/race (non-Hispanic White, non-Hispanic Black, Hispanic, non-Hispanic Asian or Pacific Islander, non-Hispanic American Indian or Alaska Native, and Hispanic [any race]), educational level (categorized as less than high school, high school or general educational development, or at least some college), and income (categorized as<$25 000, $25 000-$39 999, $40 000-$69 999, or≤$70 000).

### Statistical Analysis

Data were analyzed from December 2020 to January 2021 using SAS statistical software, version 9.4 (SAS Institute). We accounted for the NESARC-III survey design by using PROC SURVEY procedures in SAS with the Taylor series-variance-estimation method. Weighted means, frequencies, and cross tabulations were computed for prevalence of lifetime and past-year SAs, and the Rao-Scott χ^2^ test was used to compare the proportion of participants reporting lifetime and past-year SAs by lifetime BPD diagnosis and by each specific BPD criterion. The differences in prevalence estimates were compared between 4 BPD diagnostic codes, using 2-tailed *t* tests with Bonferroni corrections for multiple comparisons (significance was set at *P* < .009). Multivariable-adjusted logistic regression was used to calculate adjusted odds ratios (AORs) and 95% CIs and compared the risk of lifetime and past-year SAs by BPD diagnosis and by each specific BPD criterion. We ran 3 separate models: model 1 adjusted for sociodemographic variables; model 2 additionally adjusted for diagnosis of other psychiatric disorders (any mood disorder, any anxiety disorder, any substance use disorder, PTSD, antisocial personality disorder, schizotypal personality disorder, and conduct disorder), age at BPD onset, and a history of adverse childhood experiences (ACEs); and model 3 simultaneously entered all BPD-specific criteria except for self-injurious behavior, after adjusting for the variables in model 2. Multinomial logistic regression was used to compare the risk of 1 or multiple lifetime SAs by each specific BPD criterion, adjusting for sociodemographic variables, other psychiatric disorders, age at BPD onset, and history of ACEs. Significant Wald χ^2^ values were probed using the Tukey-Kramer post-hoc test.

## Results

### Prevalence of SA by Diagnosis of BPD

Of the 36 309 participants, 20 442 (56.3%) were women, 15 867 (43.7%) were men, 52.9% were non-Hispanic White, and the mean (SD) age was 45.6 (17.5) years. The sociodemographic characteristics, prevalence of other psychiatric disorders, and history of ACEs by each specific BPD criterion for the total sample and by SA history are summarized in eTables 2 and 3 in the Supplement.

With use of the original NESARC-III BPD diagnostic code requiring 1 criterion to meet social-occupational dysfunction, the prevalence (SE) was 22.7% (0.8%) for lifetime SAs and 2.1% (0.2%) for past-year SAs among respondents with a BPD diagnosis and 2.9% (0.1%) for lifetime SAs and 0.1% (0.0%) for past-year SAs among respondents without a BPD diagnosis (Table 1). With use of the original NESARC-III BPD diagnostic code requiring 2 criteria to meet social-occupational dysfunction, the prevalence (SE) was 23.8% (0.9%) for lifetime SAs and 2.2% (0.2%) for past-year SAs among respondents with a BPD diagnosis and 3.1% (0.1%) for lifetime SAs and 0.2% (0.0%) for past-year SAs among respondents without a BPD diagnosis. With use of our scoring procedure (ie, requiring at least 5 specific criteria to meet social-occupational dysfunction), the prevalence (SE) was 30.4% (1.1%) for lifetime SAs and 3.2% (0.4%) for past-year SAs among respondents with a BPD diagnosis and 3.7% (0.2%) for lifetime SAs and 0.2% (0.0%) for past-year SAs among respondents without a BPD diagnosis. When parallel analyses were conducted excluding the BPD criterion of self-injurious behavior (to eliminate the criterion-overlap confound), the prevalence (SE) was 28.1% (1.1%) for lifetime SAs and 3.0% (0.4%) for past-year SAs among respondents with a BPD diagnosis and 3.8% (0.2%) for lifetime SAs and 0.2% (0.0%) for past-year SAs among respondents without a BPD diagnosis. Thus, regardless of the operationalization of the BPD diagnosis, the prevalence of SAs was greater among those categorized with a BPD diagnosis than those without a BPD diagnosis. Statistical testing revealed that the prevalence of lifetime SAs was greater when using our scoring procedures than when using the NESARC-III original diagnostic codes (Table 1).

**Table 1.** Prevalence and Odds Ratios of Reporting Suicide Attempts by *DSM-5* BPD Diagnosis^a^

Criterion	Patients, No/total No. (%) [SE]^b^		Model 1^c^		Model 2^d^		
	Met BPD diagnosis	Did not meet BPD diagnosis	Wald χ^2^	AOR (95% CI)	Wald χ^2^	AOR (95% CI)
**Lifetime suicide attempts**								
SOD required for 1 criterion^e^	1010/4289 (22.7) [0.8]^f^	985/31 882 (2.9) [0.1]	1484.17^f^	8.40 (7.53-9.37)	103.94^f^	2.18 (1.88-2.53)
SOD required for 2 criteria^e^	939/3789 (23.8) [0.9]^f^	1056/32 382 (3.1) [0.1]	1216.01^f^	8.42 (7.46-9.51)	98.68^f^	2.12 (1.82-2.46)
SOD required for all criteria^g^	670/2171 (30.4) [1.1]^f^^,^^h^^,^^i^	1325/34 000 (3.7) [0.2]	1060.02^f^	9.15 (7.99-10.47)	88.19^f^	2.10 (1.79-2.45)
SOD required for all criteria but without SIB^g^	591/2060 (28.1) [1.1]^f^^,^^h^^,^^i^	1404/34 111 (3.8) [0.2]	921.22^f^	7.61 (6.67-8.69)	31.9^f^	1.59 (1.35-1.88)
**Past-year suicide attempts**								
SOD required for 1 criterion^e^	99/4289 (2.1) [0.2]^f^	53/31 882 (0.1) [0.0]	146.24^f^	11.77 (7.86-17.62)	19.16^f^	3.31 (1.92-5.68)
SOD required for 2 criteria^e^	91/3789 (2.2) [0.2]^f^	61/32 382 (0.2) [0.0]	139.68^f^	10.58 (7.13-15.72)	14.88^f^	2.83 (1.66-4.82)
SOD required for all criteria^g^	67/2171 (3.2) [0.4]^f^	85/34 000 (0.2) [0.0]	150.90^f^	11.42 (7.71-16.91)	24.13^f^	3.13 (1.97-4.95)
SOD required for all criteria but without SIB^g^	59/2060 (3.0) [0.4]^f^	93/34 111 (0.2) [0.0]	132.86^f^	9.83 (6.63-14.55)	14.79^f^	2.48 (1.56-3.97)

Abbreviations: AOR, adjusted odds ratio; BPD, borderline personality disorder; *DSM-5*, *Diagnostic and Statistical Manual of Mental Disorders* (5th edition); NESARC-III, National Epidemiological Survey on Alcohol and Related Conditions-III; SIB, self-injurious behavior; SOD, social-occupational dysfunction.

^a^ All analyses were adjusted for the NESARC complex survey design.

^b^ Prevalence percentages are weighted means.

^c^ Model 1 was adjusted for demographic characteristics (sex, age group, educational level, race, and income).

^d^ Model 2 was additionally adjusted for lifetime diagnosis of other psychiatric disorders (any mood disorder, any anxiety disorder, any substance use disorder, posttraumatic stress disorder, and other personality disorder [schizotypal personality disorder, antisocial personality disorder, or conduct disorder]), childhood adverse experiences, and age at first BPD diagnosis.

^e^ Diagnostic code provided by the NESARC-III.

^f^ Significant at *P* < .001.

^g^ Diagnostic code created by the authors (operationalization is explained in eTable 1 in the Supplement).

^h^ Significantly different from values when SOD was required for 1 criterion at *P* < .009, based on a 2-tailed *t* test with Bonferroni correction.

^i^ Significantly different from values when SOD was required for 2 criteria at *P* < .009, based on a 2-tailed *t* test with Bonferroni correction.

### Prevalence of SA by Each Specific Criterion of BPD

The prevalence of SAs by each specific BPD criterion among respondents who met criteria for BPD (requiring at least 5 specific criteria to be associated with social-occupational dysfunction) is summarized in Table 2. The prevalence (SE) of lifetime SAs was significantly greater when respondents met criteria for chronic emptiness (34.9% [1.4%] vs 20.7% [2.0%]; *P* < .001), self-injurious behavior (71.7% [2.2%] vs 9.3% [1.0%]; *P* < .001), and impulsivity (31.8% [1.4%] vs 23.2% [2.6%]; *P* = .009). The prevalence (SE) of past-year SA was significantly greater when respondents met criteria for chronic emptiness (3.9% [0.6%] vs 1.7% [0.4%]; *P* = .005) and self-injurious behavior (8.5% [1.2%] vs 0.4% [0.2%]; *P* = <.001).

**Table 2.** Prevalence and Odds Ratios of Reporting Suicide Attempts by Each Specific BPD Criterion^a^

Criterion	Patients, No./total No. (%) [SE]^b^		Model 1^c^		Model 2^d^		Model 3^e^	
	Met criterion	Did not meet criterion	Wald χ^2^	AOR (95% CI)	Wald χ^2^	AOR (95% CI)	Wald χ^2^	AOR (95% CI)
**Lifetime suicide attempts**								
Unstable relationships	563/1846 (30.4) [1.2]	107/325 (30.6) [2.8]	0.34	0.92 (0.68-1.23)	0.53	0.90 (0.67-1.21)	0.53	0.90 (0.67-1.20)
Affective instability	585/1875 (30.7) [1.2]	85/296 (28.7) [3.1]	0.07	1.05 (0.75-1.46)	0.59	0.87 (0.61-1.24)	0.52	0.88 (0.62-1.25)
Abandonment fear	493/1617 (31.0) [1.4]	177/554 (28.9) [2.3]	0.21	1.06 (0.82-1.37)	0.03	0.98 (0.74-1.29)	0.00	1.00 (0.75-1.33)
Anger	531/1722 (30.4) [1.4]	139/449 (30.5) [2.5]	0.03	0.98 (0.73-1.31)	0.24	0.93 (0.69-1.25)	0.13	0.95 (0.70-1.28)
Identity disturbance	467/1468 (30.9) [1.6]	203/703 (29.4) [1.7]	0.38	1.07 (0.86-1.35)	0.59	0.91 (0.72-1.16)	1.38	0.87 (0.67-1.11)
Emptiness	528/1476 (34.9) [1.4]^f^	142/695 (20.7) [2.0]	20.24^f^	1.91 (1.44-2.54)	8.83^g^	1.58 (1.16-2.14)	11.27^f^	1.66 (1.23-2.24)
Dissociation/paranoia	490/1530 (31.3) [1.3]	180/641 (28.4) [2.2]	0.54	1.09 (0.86-1.39)	0.40	0.92 (0.70-1.20)	1.51	0.85 (0.66-1.10)
Self-injurious behavior	525/739 (71.7) [2.2]^f^	145/1432 (9.3) [1.0]	346.44^f^	25.28 (17.92-35.66)	297.85^f^	24.28 (16.83-32.03)	NA	NA
Impulsivity	587/1819 (31.8) [1.4]^f^	83/352 (23.2) [2.6]	7.12^h^	1.55 (1.12-2.16)	1.56	1.24 (0.88-1.75)	1.81	1.27 (0.89-1.80)
**Past-year suicide attempts**								
Unstable relationships	57/1846 (3.2) [0.5]	10/325 (2.8) [0.9]	0.02	1.06 (0.51-2.21)	0.00	0.99 (0.46-2.10)	0.13	0.87 (0.42-1.83)
Affective instability	64/1875 (3.4) [0.5]	3/296 (1.7) [1.2]	1.03	2.10 (0.49-8.97)	0.59	1.73 (0.42-7.22)	0.60	1.72 (0.43-6.85)
Abandonment fear	56/1617 (3.6) [0.6]^i^	11/554 (1.8) [0.6]	3.78	2.04 (0.99-4.22)	2.77	1.82 (0.89-3.71)	3.10	1.96 (0.92-4.16)
Anger	54/1722 (3.4) [0.5]	13/449 (2.1) [0.6]	2.81	1.68 (0.91-3.13)	2.32	1.61 (0.87-2.99)	1.88	1.58 (0.82-3.05)
Identity disturbance	46/1468 (3.0) [0.5]	21/703 (3.5) [0.9]	0.20	0.86 (0.45-1.67)	1.08	0.70 (0.37-1.36)	1.64	0.64 (0.33-1.27)
Emptiness	56/1476 (3.9) [0.6]^f^	11/695 (1.7) [0.4]	8.41^j^	2.43 (1.33-4.47)	4.96^i^	1.99 (1.08-3.66)	5.91^i^	2.45 (1.18-5.08)
Dissociation/paranoia	51/1530 (3.2) [0.5]	16/641 (3.1) [0.9]	0.01	1.03 (0.54-1.94)	0.37	0.81 (0.41-1.60)	0.70	0.73 (0.35-1.53)
Self-injurious behavior	62/739 (8.5) [1.2]^f^	5/1432 (0.4) [0.2]	23.68^f^	21.64 (6.10-73.39)	20.09^f^	19.32 (5.22-71.58)	NA	NA
Impulsivity	62/1819 (3.4) [0.5]	5/352 (1.6) [0.9]	1.52	2.08 (0.64-6.29)	0.91	1.84 (0.52-6.55)	0.99	1.91 (0.53-6.86)

Abbreviations: AOR, adjusted odds ratio; BPD, borderline personality disorder; NA, not applicable; NESARC, National Epidemiological Survey on Alcohol and Related Conditions.

^a^ The analysis included respondents who met criteria for BPD with at least 5 specific criteria associated with social-occupational dysfunction. All analyses were adjusted for the NESARC complex surgery design.

^b^ Prevalence percentages are weighted means.

^c^ Model 1 was adjusted for demographic characteristics (sex, age group, educational level, race, and income).

^d^ Model 2 was additionally adjusted for lifetime diagnosis of other psychiatric disorders (any mood disorder, any anxiety disorder, any substance use disorder, posttraumatic stress disorder, and other personality disorder [schizotypal personality disorder, antisocial personality disorder, or conduct disorder]), childhood adverse experiences, and age at first BPD diagnosis.

^e^ In Model 3, BPD-specific criteria were entered in the model while simultaneously excluding self-injurious behavior.

^f^ Significant at *P* < .001.

^g^ Significant at *P* = .004.

^h^ Significant at *P* = .009.

^i^ Significant at *P* < .05.

^j^ Significant at *P* = .005.

In the model adjusted for sociodemographic characteristics, 3 BPD criteria were significantly associated with increased odds of lifetime SAs: chronic emptiness (AOR, 1.91; 95% CI, 1.44-2.54), self-injurious behavior (AOR, 25.28; 95% CI, 17.92-35.66), and impulsivity (AOR, 1.55; 95% CI, 1.12-2.16). Chronic emptiness and self-injurious behaviors were also significantly associated with increased odds of past-year SAs (emptiness: AOR, 2.43; 95% CI, 1.33-4.47; self-injury: AOR, 21.64; 95% CI, 6.10-73.39) (Table 2). In the model adjusting additionally for other psychiatric disorders, age at first BPD onset, and history of ACEs, 2 BPD criteria remained significantly associated with increased odds of lifetime and past-year SA: chronic emptiness (lifetime: AOR, 1.58; 95% CI, 1.16-2.14; past year: AOR, 1.99; 95% CI, 1.08-3.66) and self-injurious behavior (lifetime: AOR, 24.28; 95% CI, 16.83-32.03; past year: AOR, 19.32; 95% CI, 5.22-71.58). When all specific BPD criteria were simultaneously entered (excluding self-injurious behavior), chronic emptiness was significantly associated with increased odds for lifetime SAs (AOR, 1.66; 95% CI, 1.23-2.24) and past-year SAs (AOR, 2.45; 95% CI, 1.18-5.08) (Table 2).

### Number of SAs and Specific Criteria of BPD

The prevalence of 0, single, and multiple lifetime SAs by each specific BPD criterion is summarized in Table 3. The prevalence (SE) of 2 or more SAs was significantly greater among participants meeting criteria for emptiness (18.4% [1.1%] vs 7.6% [1.3%]; *P* < .001), self-injurious behaviors (39.3% [2.1%] vs 2.9% [0.6%]; *P* < .001), and impulsivity (16.2% [1.0%] vs 8.7% [1.5%]; *P* < .001) than among those who did not meet these criteria. The prevalence (SE) of 1 SA was also significantly greater among participants who met criteria for self-injurious behaviors than among those who did not (31.7% [2.0%] vs 6.4% [0.7%]; *P* < .001). In the analysis adjusted for sociodemographic characteristics, other psychiatric disorders, age at first BPD onset, and ACEs, emptiness and self-injurious behaviors were significantly associated with increased odds of reporting multiple SAs compared with reporting no SAs (emptiness: AOR, 2.15; 95% CI, 1.41-3.29; self-injury: AOR, 40.38; 95% CI, 23.62-69.03). Self-injurious behavior was also significantly associated with increased odds of reporting a single SA compared with no SAs (AOR, 16.08; 95% CI, 11.16-23.17) as well as reporting multiple SAs compared with a single SA (AOR, 2.51; 95% CI, 1.53-4.11).

**Table 3.** Risk for 1 or Multiple Suicide Attempts in Lifetime by Each Specific BPD Criterion^a^

Criterion	Patients, % (SE)^b^						Wald χ^2^	AOR (95% CI)		
	Met specific BPD criterion			Did not meet specific BPD criterion				1 SA vs 0 SAs	≥2 SAs vs 0 SAs	≥2 SAs vs 1 SA
	0 SAs	1 SA	≥2 SAs	0 SAs	1 SA	≥2 SAs				
Unstable relationships	70.3 (1.2)	14.8 (1.0)	14.9 (0.9)	69.8 (2.9)	14.8 (2.3)	15.4 (2.1)	0.43	0.94 (0.64-1.38)	0.83 (0.56-1.23)	0.89 (0.55-1.43)
Affective instability	69.9 (1.2)	15.4 (1.0)	14.7 (0.9)	71.9 (3.1)	11.2 (2.0)	16.9 (2.9)	2.24	1.20 (0.78-1.83)	0.63 (0.38-1.05)	0.53 (0.29-0.97)
Abandonment fears	69.5 (1.4)	15.4 (1.1)	15.1 (1.0)	72.1 (2.3)	13.3 (1.5)	14.6 (1.8)	0.46	1.10 (0.80-1.52)	0.92 (0.63-1.34)	0.83 (0.56-1.23)
Anger	70.3 (2.5)	14.3 (1.9)	15.3 (1.9)	70.1 (1.4)	14.9 (1.2)	14.9 (1.0)	0.39	1.02 (0.68-1.52)	0.87 (0.62-1.22)	0.86 (0.56-1.32)
Identity disturbance	69.6 (1.6)	15.6 (1.2)	14.8 (1.1)	71.4 (1.7)	13.1 (1.3)	15.4 (1.5)	1.33	1.07 (0.79-1.44)	0.80 (0.58-1.10)	0.75 (0.52-1.09)
Emptiness^c^	65.7 (1.4)	15.9 (1.2)	18.4 (1.1)^d^	80.0 (2.0)	12.4 (1.8)	7.6 (1.3)	6.85^e^	1.32 (0.87-2.00)	2.15 (1.41-3.29)^d^	1.63 (0.93-2.89)
Dissociation or paranoia	69.3 (1.3)	15.0 (1.1)	15.7 (1.1)	72.2 (2.1)	14.4 (1.7)	13.4 (1.7)	0.18	0.92 (0.65-1.30)	0.92 (0.63-1.34)	1.00 (0.63-1.59)
Self-injurious behaviors^c^	29.0 (2.3)	31.7 (2.0)^d^	39.3 (2.1)^d^	90.7 (1.0)	6.4 (0.7)	2.9 (0.6)	141.73^d^	16.08 (11.16-23.17)^d^	40.38 (23.62-69.03)^d^	2.51 (1.53-4.11)^d^
Impulsivity^c^	68.9 (1.4)	14.9 (1.0)	16.2 (1.0)^d^	77.0 (2.5)	14.3 (2.2)	8.7 (1.5)	2.44	1.00 (0.66-1.50)	1.57 (1.03-2.41)^f^	1.58 (0.97-2.59)

Abbreviations: AOR, adjusted odds ratio; BPD, borderline personality disorder; NESARC, National Epidemiological Survey on Alcohol and Related Conditions; SA, suicide attempt.

^a^ All analyses were adjusted for the NESARC complex survey design and for demographic characteristics (sex, age group, educational level, race, and income), lifetime diagnosis of other psychiatric disorders, childhood adverse experiences, and age at first BPD diagnosis.

^b^ Prevalence percentages are weighted means.

^c^ Rao-Scott χ^2^ analysis was significant at *P* < .05.

^d^ Significant at *P* < .001.

^e^ Significant at *P* = .002.

^f^ Significant at *P* = .04 but not reported as Wald χ^2^ because the omnibus test of impulsivity was not significant at *P* < .05.

## Discussion

This cross-sectional study examined the lifetime and past-year prevalence of SAs in a representative national sample of US adults with lifetime BPD using data from the 2012-2013 NESARC-III and found that lifetime and past-year SAs were common in US adults with lifetime BPD. With use of the original NESARC-III BPD diagnostic code requiring 1 criterion to meet social-occupational dysfunction, the lifetime prevalence of SAs was 22.7% among those with BPD vs 2.9% among those without BPD. With use of the original NESARC-III BPD diagnostic code requiring 2 criteria to meet social-occupational dysfunction, the lifetime prevalence of SAs was 23.8% among those with BPD vs 3.1% among those without a BPD diagnosis. With use of our scoring procedure, which required 5 specific criteria to be associated with social-occupational dysfunction, the lifetime prevalence of SAs was 30.4% among those with BPD and 3.7% among those without BPD. Even when excluding the BPD criterion of self-injurious behavior (to eliminate the criterion-overlap confound), the lifetime prevalence of SAs was 28.1% among those with BPD vs 3.8% among those without BPD. Analyses revealed that the prevalence rates of lifetime SAs associated with BPD when using our scoring procedures were significantly higher than when using the original NESARC-III diagnostic codes. This study’s findings suggest that lifetime and past-year SAs may be common among persons with lifetime BPD, even when excluding the criterion of self-injurious behaviors, and that social-occupational dysfunction associated with BPD criteria may be associated with increased risk for SAs.

After adjusting for demographic and other clinical variables, a BPD diagnosis and the specific criteria of self-injurious behaviors and chronic feelings of emptiness were significantly associated with elevated risk for lifetime and past-year history of SAs. Chronic feelings of emptiness remained significantly associated with elevated risk for lifetime and past-year SAs after controlling for all other BPD criteria as well. These findings are consistent with those of prior work with samples of treatment-seeking individuals that documented significant associations between BPD and self-injurious behaviors^9,13^ and between chronic emptiness and increased risk for SAs.^13^ These findings are complementary to those of a previous NESARC-III study reporting that among respondents with recent SAs, a significantly elevated rate of BPD was observed alongside elevated rates of other psychiatric disorders.^4^ Of note, the analysis by Olfson and colleagues^4^ of the complementary question concerning the association of SA with BPD relied on original NESARC-III BPD scoring (which, as shown in the present study, may produce divergent odds ratios) and did not consider BPD criteria.

This study’s findings regarding the significant association between the specific BPD criterion of chronic feelings of emptiness and increased SA risk are consistent with findings reported for a treatment-seeking clinical sample followed up over 10 years.^13^ However, in contrast to Yen and colleagues,^13^ we did not observe significant associations for 2 other specific criteria (identity disturbance and frantic attempts to avoid abandonment). This cross-sectional study retrospectively assessed lifetime and past-year SAs, whereas the study by Yen and colleagues^13^ was based on 10 years of prospective follow-up to capture SA data. Thus, the convergence between this study’s findings and those of Yen and colleagues^13^ is important because of the complementary epidemiological and clinical (treatment-seeking) sampling methods and the longer-term perspectives afforded by this study’s lifetime data and the 10-year prospective data analyzed by Yen and colleagues.^13^

This study’s findings are supported by a sample size of persons with lifetime BPD (ranging from 2171 to 4289 depending on the strictness of diagnostic coding) that was much larger than that in previous research. The larger sample reduces SEs and the width of 95% CIs, and the epidemiological sampling enhances generalizability by reducing sampling confounds associated with clinical samples of treatment-seeking individuals with high comorbidity rates.^19,20,21,22^ Thus, we highlight the potential generalizability of findings that chronic feelings of emptiness are associated with increased SA risk. Chronic emptiness, although under-recognized and under-researched in BPD compared with more florid features such as affective instability, impulsivity, and self-injurious behaviors,^35^ is included as part of the personality functioning dimension of the Alternative Model for Personality Disorders in Section III of the *DSM-5*.^14^ Chronic emptiness, which is distinct from related constructs of loneliness and hopelessness, reflects a sense of disconnection from both self and others,^35^ is associated with poorer treatment and functional outcomes,^36^ and is central in prominent conceptual models of SAs.^37,38^

This study’s findings have implications for future practice and research. Clinically, the findings suggest the value of asking about chronic feelings of emptiness in addition to queries about self-injurious behaviors during suicide risk screenings and assessments in clinical and nonclinical settings. Individuals performing screening and clinicians might, for example, begin with asking questions based on the AUDADIS-5^28^ interview used in the NESARC-III (eg, “Have you often felt like your life had no purpose or meaning?” or “Have you often felt empty inside?”) to assess chronic emptiness (eTable 1 in the Supplement). Future research should develop improved screening and psychometrically sound measures of chronic emptiness and test their prognostic usefulness in naturalistic and treatment-outcome studies.^35^

### Limitations

This study has limitations. Reliance on retrospective self-report and possible recall biases and the use of lay, albeit experienced and trained, interviewers are issues characteristic of large-scale epidemiological studies and have been previously discussed.^39^ Sampling, although reasonably representative of US adults, did not include institutionalized, incarcerated, or homeless persons, which are groups with known high rates of suicide and self-harm behaviors.^40^ The severity and duration of BPD and other criteria were not evaluated, and therefore, potential associations with SAs could not be explored; we were, however, able to statistically adjust for age at BPD onset, which did not alter the findings. In addition, because of the cross-sectional nature of the analyses, causality could not be assessed.

## Conclusions

In this cross-sectional study of a nationally representative sample of US adults, after adjusting for demographic and other clinical variables, lifetime BPD and the specific criteria of self-injurious behaviors and chronic feelings of emptiness were significantly associated with elevated risk for SAs. The findings suggest that although BPD is a complex diagnostic construct, the specific BPD criteria of self-injurious behaviors and chronic feelings of emptiness should be routinely considered in suicide risk screenings and assessments in clinical and nonclinical settings.

## Notes

Supplement. **eTable 1.** Operationalization of specific criteria for *DSM-5* borderline personality disorder (BPD) for the current study **eTable 2.** Frequency and percentage meeting specific borderline personality disorder (BPD) criteria by demographic characteristics in the total sample and by SA history **eTable 3.** Prevalence of lifetime psychiatric disorders and childhood adversity by SA history and by specific borderline personality disorder (BPD) criteria
