# Supplementary material for: Association of Borderline Personality Disorder Criteria With Suicide Attempts Among US Adults
Source: JAMA Netw Open. 2021 May 11;4(5):e219389. doi: 10.1001/jamanetworkopen.2021.9389 (PMC8114135; doi:10.1001/jamanetworkopen.2021.9389)
Supplement: Supplement. — eTable 1. Operationalization of specific criteria for DSM-5 borderline personality disorder (BPD) for the current study eTable 2. Frequency and percentage meeting specific borderline personality disorder (BPD) criteria by demographic characteristics in the total sample and by SA history eTable 3. Prevalence of lifetime psychiatric disorders and childhood adversity by SA history and by specific borderline personality disorder (BPD) criteria [file jamanetwopen-e219389-s001.pdf]

Supplementary Online Content

Grilo CM, Udo T. Association of borderline personality disorder criteria with suicide attempts among US adults. *JAMA Netw Open*. 2021;4(5):e219389. doi:10.1001/jamanetworkopen.2021.9389

eTable 1. Operationalization of specific criteria for *DSM-5* borderline personality disorder (BPD) for the current study

eTable 2. Frequency and percentage meeting specific borderline personality disorder (BPD) criteria by demographic characteristics in the total sample and by SA history

eTable 3. Prevalence of lifetime psychiatric disorders and childhood adversity by SA history and by specific borderline personality disorder (BPD) criteria

This supplementary material has been provided by the authors to give readers additional information about their work.

© 2021 Grilo CM et al. *JAMA Network Open*.

eTable 1. Operationalization of specific criteria for *DSM-5* Borderline Personality Disorder (BPD) for the current study.

***DSM-5* Criteria**	**Item number**	**The AUDADIS-5 questions used in this study**
1) **Abandonment fears**: Frantic efforts to avoid real or imagined abandonment.<br><br>*Note*: Do not include suicidal or self-mutilating behavior covered in Criterion 5.	N10Q1A7	When you have gotten close to someone, have you needed them to reassure you that they would never leave you?
	N10Q1A8	Have you put a lot of time and effort into doing things to keep someone from leaving you?
	N10Q1A9	Have you often become frantic when you thought that someone you really cared about was going to leave you?
	N10Q1A10	Have you gone to extremes to keep people from leaving you?
2) **Unstable relationships**: A pattern of unstable and intense interpersonal relationships characterized by alternating between extremes of idealization and devaluation	N10Q1A1	Have you usually gotten attached to people very quickly?
	N10Q1A2	Have your relationships with people you really cared about had lots of extreme ups and downs?
	N10Q1A3	Have you often started out thinking that someone was a great person only to be disappointed when they didn't live up to your expectations?
3) **Identity disturbance**: Markedly and persistently unstable self-image or sense of self	N10Q1A14	Have there been lots of sudden changes in your personal goals, career plans, religious beliefs, or other important aspects of your life?
	N10Q1A15	Have you been so different with different people or in different situations that you sometimes don't know who you really are?
	N10Q1A16	Has your sense of who you are often changed depending on the situation or whom you are with?
	N10Q1A17	Have you all of a sudden changed your sense of who you are and where you are headed?

© 2021 Grilo CM et al. *JAMA Network Open*.

DSM-5 Criteria	Item number	The AUDADIS-5 questions used in this study
4) **Impulsivity**: In at least two areas that are potentially self-damaging (e.g., spending, sex, substance abuse, reckless driving, binge eating).<br><br>*Note*: Do not include suicidal or self-mutilating behavior covered in Criterion 5.	N10Q1A25<br><br>N10Q1A26<br><br>N10Q1A27<br><br>N10Q1A28<br><br>N10Q1A29<br><br>N10Q1A30	Have you gotten into sexual relationships quickly or without thinking about the consequences?<br><br>Have there been periods of your life when you often spent too much money while shopping or gambling?<br><br>Have you had periods in your life when you drank a lot more or used a lot more drugs than you meant to?<br><br>Have you had periods in your life when you often took too many risks when driving?<br><br>Have you often done things impulsively, not caring about the consequences?<br><br>Have you often engaged in reckless behavior without thinking about how dangerous it could be?
5) **Self-injurious behaviors**: recurrent suicidal behavior, gestures, or threats, or self-mutilating behavior	N10Q1A23<br><br>N10Q1A24	When you've been under a lot of stress, have you cut, burned, or scratched yourself on purpose?<br><br>Have you tried to hurt or kill yourself, or threatened to do so?
6) **Affective instability**: Due to a marked reactivity of mood (e.g., intense episodic dysphoria, irritability, or anxiety usually lasting a few hours and only rarely more than a few days)	N10Q1A4<br><br>N10Q1A5<br><br>N10Q1A6	Have you often become very sad, anxious or angry over little things?<br><br>Have other people often wondered why you get so upset so easily?<br><br>Have you had a lot of sudden mood changes?
7) **Emptiness**: Chronic feelings of emptiness	N10Q1A18<br><br>N10Q1A19	Have you often felt like your life had no purpose or meaning?<br><br>Have you often felt empty inside?

© 2021 Grilo CM et al. *JAMA Network Open*.

DSM-5 Criteria	Item number	The AUDADIS-5 questions used in this study
8) **Anger**: Inappropriate, intense anger or difficulty controlling anger (e.g., frequent displays of temper, constant anger, recurrent physical fight)	N10Q1A11<br><br>N10Q1A12<br><br>N10Q1A13	Have you often had temper outbursts or gotten so angry that you lose control?<br><br>Have you hit people or thrown things when you got angry?<br><br>Have even little things made you angry or have you had difficulty controlling your anger?
9) **Dissociation/paranoia**: Transient, stress-related paranoid ideation or severe dissociative symptoms	N10Q1A20<br><br>N10Q1A21<br><br>N10Q1A22	When you've been under a lot of stress, have you often felt that you weren't real?<br><br>When you've been under a lot of stress, have you often felt like you were outside your body?<br><br>When you've been under a lot of stress, have you felt suspicious or distrustful of other people?
**Social-occupational dysfunction**	N10Q1B1-N10Q1B30	This question was asked each time respondent endorsed any of the above questions.<br><br>Did this ever trouble you or cause problems at work, school, or with your family or other people?

© 2021 Grilo CM et al. *JAMA Network Open*.

eTable 2. Frequency and percentage meeting specific BPD criteria by demographic characteristics in the total sample and by SA history.

	Unstable Relationships		Affective Instability		Abandonment Fears		Anger		Identify Disturbance		Emptiness		Dissociation/ Paranoia		Self-injurious Behaviors		Impulsivity	
	n	% (SE)	n	% (SE)	n	% (SE)	n	% (SE)	n	% (SE)	n	% (SE)	n	% (SE)	n	% (SE)	n	% (SE)
**Total (N = 2171)**	1853	83.9 (0.89)	1882	86.8 (0.85)	1623	73.8 (1.25)	1730	80.1 (0.97)	1476	67.3 (1.36)	1483	68.4 (1.22)	1537	69.7 (1.34)	741	33.8 (1.22)	182 7	84.2 (0.90)
Sex																		
Men (n = 834)	714	82.5 (1.62)	701	84.9 (1.46)	623	74.2 (1.80)	678	83.6 (1.39)[‡]	561	67.0 (1.85)	540	64.8 (2.02)[†]	565	67.6 (2.14)	243	29.7 (2.07)[‡]	721	86.4 (1.34)[†]
Women (n = 1137)	1139	84.9 (1.17)	1181	88.1 (1.24)	1000	73.5 (1.80)	1052	77.5 (1.52)	915	67.6 (1.73)	943	71.1 (1.47)	972	71.2 (1.51)	498	36.8 (1.58)	110 6	82.6 (1.17)
Race or ethnicity																		
Non-Hispanic White (n = 1248)	1051	83.2 (1.13)	1076	86.6 (1.08)	929	73.6 (1.70)	986	80.0 (1.36)	839	67.6 (1.70)	875	69.5 (1.27)	851	67.9 (1.62)	451	34.7 (1.57)	106 2	85.0 (1.10)
Non-Hispanic Black (n = 436)	376	84.9 (2.15)	384	86.2 (2.34)	327	73.1 (2.80)	357	81.1 (2.04)	311	68.9 (3.13)	281	64.6 (2.80)	335	77.4 (2.45) †	134	29.4 (2.40)	360	81.9 (2.53)
Hispanic (n = 388)	339	85.2 (1.90)	336	87.9 (1.83)	287	74.0 (2.36)	305	78.4 (2.37)	259	67.1 (2.44)	251	63.0 (2.86)	277	71.0 (2.42)	118	31.9 (2.88)	320	80.6 (2.61)
Other [1] (n = 99)	87	88.4 (3.87)	86	87.8 (3.48)	80	76.8 (4.63)	82	82.5 (4.72)	67	61.2 (5.96)	76	77.0 (4.60)[†]	74	73.1 (5.21)	38	36.8 (4.64)	85	87.0 (3.41)
Age (years)																		
18-29 (n = 553)	486	88.3 (1.67)	491	88.8 (1.75)	421	73.1 (3.02)	448	81.2 (1.97)	365	66.4 (2.40)	351	64.7 (2.88)	370	67.9 (2.92)	206	39.5 (2.77)[‡]	468	85.0 (1.79)
30-44 (n = 669)	598	83.9 (1.30)	613	87.5 (1.54)	518	74.4 (1.96)	574	83.2 (1.64)	469	65.0 (2.39)	471	67.9 (1.90)	499	68.4 (2.07)	230	31.6 (1.75)	599	85.0 (1.44)
45-59 (n = 662)	560	82.8 (1.70)	566	86.2 (1.56)	498	74.3 (2.11)	509	77.9 (1.77)	463	68.3 (2.50)	478	71.8 (2.39)	486	72.6 (2.08)	241	35.2 (2.14)	553	84.1 (1.47)
≥ 60 (n = 257)	209	77.3 (3.24)[‡]	212	81.8 (2.70)	186	72.4 (3.14)	199	75.4 (3.33)	179	72.6 (3.25)	183	69.6 (2.84)	182	69.1 (2.79)	64	23.4 (2.98)	207	80.9 (2.84)
Education level																		
Less than high school (n = 363)	314	82.7 (2.70)	320	87.4 (2.19)	281	73.6 (2.99)	303	84.1 (2.08)	257	71.1 (3.20)	266	74.2 (2.72)	282	78.8 (2.77)[‡]	142	41.5 (2.99)[†]	304	83.7 (2.40)
High school or GED (n = 699)	595	85.0 (1.74)	614	88.0 (1.38)	531	74.8 (2.00)	575	82.9 (1.74)	477	65.9 (2.30)	494	68.8 (2.29)	503	70.0 (2.02)	237	32.9 (2.29)	571	81.4 (1.76)
Some college or higher (n = 1109)	944	83.7 (1.25)	948	85.8 (1.28)	811	73.2 (1.72)	852	76.9 (1.51)[‡]	742	67.0 (1.91)	723	66.3 (1.63)	752	66.5 (1.97)	362	31.9 (1.65)	952	86.2 (1.13)

© 2021 Grilo CM et al. *JAMA Network Open*.

	Unstable Relationships		Affective Instability		Abandonment Fears		Anger		Identify Disturbance		Emptiness		Dissociation/ Paranoia		Self-injurious Behaviors		Impulsivity	
	n	% (SE)	n	% (SE)	n	% (SE)	n	% (SE)	n	% (SE)	n	% (SE)	n	% (SE)	n	% (SE)	n	% (SE)
Income level																		
<$25,000 (n = 930)	799	85.6 (1.39)	798	85.5 (1.56)	710	75.3 (1.65)	751	81.8 (1.58)	650	68.1 (2.07)	675	72.2 (2.16)[†]	684	72.2 (2.10)	366	39.5 (2.20)[‡]	789	85.4 (1.36)
$25,000-39,999 (n = 514)	447	84.8 (1.91)	457	88.5 (1.72)	392	76.3 (2.46)	399	76.9 (2.15)	362	70.4 (2.47)	358	70.8 (2.49)	356	70.8 (2.41)	185	37.2 (2.51)	423	81.4 (1.98)
$40,000-69,999 (n = 481)	407	83.0 (2.05)	421	88.6 (1.67)	348	72.5 (2.70)	385	80.1 (2.32)	314	66.0 (2.72)	300	64.1 (2.51)	331	66.8 (2.60)	132	27.1 (2.36)	393	81.3 (1.82)
≥$70,000 (n = 246)	200	80.4 (2.71)	206	84.1 (2.26)	173	68.8 (31.7)	195	80.5 (2.89)	150	63.2 (3.10)	150	63.7 (3.49)	166	67.0 (3.20)	58	26.7 (2.98)	222	90.3 (2.10)[‡]
**With SA History**																		
Sex																		
Men (n = 217)	187	84.8 (2.40)	180	83.9 (2.98)	161	76.4 (3.43)	175	81.9 (2.74)	155	69.2 (3.84)	169	76.0 (3.77)	154	69.8 (3.83)	170	80.7 (3.85)	195	90.9 (2.34)
Women (n = 453)	376	83.4 (2.46)	405	89.6 (1.95)	332	74.4 (2.84)	356	78.9 (2.40)	312	67.9 (2.72)	359	79.8 (2.49)	336	72.8 (2.61)	355	79.3 (2.49)	392	86.3 (2.06)
Race or ethnicity																		
Non-Hispanic White (n = 393)	334	85.3 (2.17)	344	88.3 (1.73)	299	77.4 (2.60)	315	81.8 (2.21)	273	69.0 (2.59)	317	79.5 (2.44)	277	70.0 (2.71)	320	81.7 (2.57)	345	87.3 (2.03)
Non-Hispanic Black (n = 125)	101	80.0 (4.69)	110	82.6 (6.69)	86	67.5 (6.47)	97	75.9 (4.52)	92	69.1 (6.89)	96	76.0 (5.31)	99	79.4 (4.62)	90	69.6 (5.55)	103	86.1 (3.67)
Hispanic (n = 111)	95	81.9 (4.58)	95	88.5 (3.41)	79	73.1 (4.04)	86	75.1 (5.11)	74	67.3 (5.95)	81	72.4 (3.79)	82	72.8 (4.41)	85	81.0 (3.85)	101	90.3 (3.82)
Other[1] (n = 41)	33	78.9 (7.82)	36	86.2 (7.02)	29	67.5 (9.42)	33	76.3 (8.46)	28	62.5 (9.02)	34	82.8 (7.51)	32	75.6 (7.52)	30	74.5 (8.74)	38	93.2 (4.38)
Age (years)																		
18-29 (n = 163)	139	85.5 (3.42)	146	88.0 (3.79)	123	74.5 (4.96)	132	82.3 (3.68)	111	67.4 (4.53)	126	79.2 (3.65)	110	70.9 (4.31)	137	86.3 (2.85)	149	91.8 (2.69)
30-44 (n = 230)	191	84.6 (2.37)	202	88.6 (2.22)	165	75.5 (3.67)	191	83.4 (2.91)	160	68.0 (4.61)	175	73.9 (3.15)	167	68.3 (3.79)	175	75.8 (4.01)	201	88.4 (2.85)
45-59 (n = 224)	184	80.5 (3.16)	196	88.0 (2.12)	168	76.5 (4.00)	171	78.6 (3.07)	160	69.3 (4.38)	187	83.8 (3.21)	175	77.7 (2.63)	175	79.6 (3.16)	189	84.4 (2.84)
≥ 60 (n = 53)	49	89.5 (5.94)	41	79.8 (5.81)	37	70.2 (7.56)	37	63.4 (7.39)[†]	36	69.4 (7.86)	40	72.5 (7.35)	38	64.0 (7.85)	38	74.5 (7.39)	48	86.5 (5.90)

© 2021 Grilo CM et al. *JAMA Network Open*.

	Unstable Relationships		Affective Instability		Abandonment Fears		Anger		Identify Disturbance		Emptiness		Dissociation/ Paranoia		Self-injurious Behaviors		Impulsivity	
	n	% (SE)	n	% (SE)	n	% (SE)	n	% (SE)	n	% (SE)	n	% (SE)	n	% (SE)	n	% (SE)	n	% (SE)
Education level																		
Less than high school (n = 128)	109	84.1 (4.61)	117	91.2 (4.13)	92	66.9 (5.85)	111	89.4 (3.14)	94	70.7 (5.57)	104	82.2 (4.37)	102	77.5 (4.83)[†]	104	84.3 (3.97)	112	88.4 (3.56)
High school or GED (n = 205)	174	84.6 (3.43)	186	90.5 (2.29)	153	75.6 (3.56)	171	82.2 (3.32)	150	69.3 (3.11)	166	79.9 (3.15)	163	77.1 (2.93)	165	80.4 (3.23)	173	85.4 (3.29)
Some college or higher (n = 337)	280	83.4 (2.39)	282	84.2 (2.21)	248	73.2 (1.72)	249	74.9 (2.84)[†]	223	66.8 (3.61)	258	76.1 (3.22)	225	66.0 (3.11)	256	77.7 (2.87)	302	89.4 (1.89)
Income level																		
<$25,000 (n = 347)	298	87.3 (1.99)	302	86.7 (2.18)	257	74.0 (3.29)	287	84.0 (2.31)	249	70.0 (3.43)	278	80.8 (2.43)	262	74.3 (3.38)	282	82.1 (2.91)	300	87.6 (2.50)
$25,000-39,999 (n = 151)	126	83.7 (3.29)	132	88.7 (3.23)	114	79.5 (4.26)	111	75.5 (3.93)	106	66.5 (4.22)	123	80.5 (3.56)	105	68.6 (4.43)	115	80.2 (3.95)	137	89.7 (2.66)
$40,000-69,999 (n = 116)	93	79.2 (4.66)	102	90.3 (2.57)	82	73.5 (4.82)	89	77.7 (4.09)	73	64.9 (4.97)	85	72.2 (4.60)	83	71.2 (5.06)	87	76.2 (3.68)	98	83.2 (4.41)
≥$70,000 (n = 56)	46	80.1 (6.05)	49	84.3 (5.61)	40	73.4 (6.14)	44	78.1 (5.38)	39	71.5 (6.21)	42	76.4 (5.46)	40	69.8 (6.61)	41	76.9 (5.87)	52	93.0 (3.75)
**Without SA History**																		
Sex																		
Men (n = 617)	524	82.0 (1.94)	517	85.2 (1.77)	459	73.6 (2.16)	498	84.1 (1.49)[†]	402	66.0 (2.30)	367	60.7 (2.26)[†]	408	66.9 (2.52)	73	12.1 (1.62)	521	84.7 (1.57)
Women (n = 884)	759	85.7 (1.29)	773	87.4 (1.60)	665	73.0 (1.99)	693	76.7 (2.00)	599	67.4 (2.15)	581	66.7 (2.00)	632	70.3 (1.77)	141	15.1 (1.42)	711	80.7 (1.48)
Race or ethnicity																		
Non-Hispanic White (n = 855)	715	82.3 (1.41)	729	85.8 (1.29)	628	72.0 (1.88)	668	79.2 (1.69)	563	66.8 (2.04)	555	65.0 (1.94)	572	67.0 (1.91)	130	14.0 (1.55)	714	84.0 (1.34)
Non-Hispanic Black (n = 311)	272	87.1 (2.43)	271	87.8 (2.21)	238	75.3 (3.13)	257	83.3 (2.39)	215	68.4 (3.46)	182	60.0 (3.54)	233	76.6 (2.95)	43	13.1 (1.81)	254	80.3 (2.81)
Hispanic (n = 277)	243	86.5 (2.14)	240	87.6 (2.37)	207	74.2 (2.48)	218	79.7 (2.61)	184	66.9 (2.90)	170	59.5 (3.49)	194	70.2 (3.26)	33	12.9 (2.38)	218	76.8 (3.33)
Other[1] (n = 58)	53	94.1 (3.10)[†]	50	89.7 (3.94)	51	83.3 (6.25)	48	81.2 (4.92)	39	61.0 (8.69)	41	73.2 (5.71)	41	71.2 (7.11)	8	13.9 (7.84)	46	83.1 (4.43)

© 2021 Grilo CM et al. *JAMA Network Open*.

	Unstable Relationships		Affective Instability		Abandonment Fears		Anger		Identify Disturbance		Emptiness		Dissociation/ Paranoia		Self-injurious Behaviors		Impulsivity	
	n	% (SE)	n	% (SE)	n	% (SE)	n	% (SE)	n	% (SE)	n	% (SE)	n	% (SE)	n	% (SE)	n	% (SE)
Age (years)																		
18-29 (n = 390)	346	89.5 (1.91)	344	89.2 (2.11)	297	72.4 (3.13)	315	80.7 (2.41)	253	65.9 (2.84)	225	58.3 (3.51)	259	66.4 (3.57)	69	18.4 (2.79)†	318	81.8 (2.18)
30-44 (n = 469)	402	83.6 (1.72)	406	87.1 (2.03)	349	74.2 (2.27)	377	82.9 (1.89)	304	63.4 (2.63)	291	64.7 (2.39)	327	68.4 (2.37)	54	10.1 (1.44)	392	81.2 (1.57)
45-59 (n = 438)	375	83.9 (2.29)	370	85.5 (2.00)	330	73.4 (2.45)	338	77.6 (2.42)	302	67.8 (2.71)	290	66.1 (2.80)	310	70.2 (2.84)	65	14.4 (1.92)	364	84.0 (1.88)
≥ 60 (n = 204)	160	74.9 (3.64)‡	170	82.1 (3.20)	148	72.7 (3.49)	161	78.1 (3.91)	142	73.2 (3.45)	142	68.7 (2.94)	144	70.9 (3.27)	26	11.1 (2.60)	158	79.4 (2.88)
Education level																		
Less than high school (n = 235)	202	81.9 (3.18)	201	85.7 (3.01)	188	78.2 (3.75)	189	81.1 (2.81)	160	71.1 (3.22)	158	69.4 (3.79)	177	79.5 (3.30)‡	37	18.3 (2.61)	189	81.1 (2.92)
High school or GED (n = 494)	418	85.1 (2.07)	425	86.9 (1.70)	375	74.4 (2.28)	401	83.1 (1.97)	324	64.4 (2.95)	326	64.3 (2.71)	337	66.9 (2.54)	71	13.3 (1.89)	395	79.7 (1.84)
Some college or higher (n = 772)	663	83.9 (1.45)	664	86.4 (1.57)	561	71.1 (2.08)	601	77.7 (1.97)	517	67.0 (2.26)	464	62.2 (1.97)	526	66.8 (2.15)	106	12.8 (1.51)	648	84.8 (1.42)
Income level																		
<$25,000 (n = 583)	496	84.4 (1.81)	493	85.0 (2.02)	450	76.2 (1.79)	460	80.5 (2.08)	397	66.9 (2.44)	394	67.1 (3.25)	417	70.7 (2.93)	83	14.2 (1.70)	485	84.1 (1.53)‡
$25,000-39,999 (n = 363)	319	85.8 (2.14)	321	88.3 (1.92)	275	75.0 (2.95)	284	77.3 (2.62)	252	71.9 (2.80)	231	66.0 (3.03)	249	72.2 (2.84)	69	18.1 (2.73)	282	77.4 (2.35)
$40,000-69,999 (n = 365)	314	84.2 (2.17)	319	88.1 (1.91)	266	72.1 (2.86)	296	80.9 (2.84)	241	66.3 (2.94)	215	61.5 (2.82)	248	65.4 (3.02)	45	11.8 (1.82)	295	80.7 (2.18)
≥$70,000 (n = 190)	154	80.5 (3.10)	157	84.0 (2.44)	133	67.3 (4.08)	151	81.3 (3.37)	111	60.5 (3.79)	108	59.5 (4.09)	126	66.1 (3.60)	17	10.5 (3.17)	170	89.4 (2.33)

Notes. All analyses were adjusted for the NESARC complex survey design. [1] = Other included Asian, Native Hawaiian, or other Pacific Islander, and Native American. † = significantly different between respondents reporting a specific criterion and who did not at $p < .05$; ‡ = significantly different between respondents reporting a specific criterion and who did not at $p < .01$.

© 2021 Grilo CM et al. *JAMA Network Open*.

eTable 3. Prevalence of lifetime psychiatric disorders and childhood adversity by lifetime SA history and by specific BPD criteria.

	With lifetime SA History				Without lifetime SA History			
	Met specific BPD criteria		Did not meet specific BPD criteria		Met specific BPD criteria		Did not meet specific BPD criteria	
	n/N	% (SE)	n/N	% (SE)	n/N	% (SE)	n/N	% (SE)
**Any anxiety disorders**								
Unstable Relationship	395/563	71.1 (2.25)	67/107	62.6 (5.19)	721/1283	59.3 (1.52)	121/218	57.9 (3.68)
Affective Instability	415/585	72.2 (2.32)‡	47/85	52.3 (6.70)	738/1290	60.4 (1.73)†	104/211	50.2 (4.06)
Abandonment Fear	351/493	71.1 (2.36)	111/177	65.6 (4.30)	631/1124	59.0 (1.63)	211/377	59.0 (3.33)
Anger	367/531	69.2 (2.47)	95/139	71.7 (4.52)	664/1191	58.1 (1.65)	178/310	62.6 (3.43)
Identity Disturbance	337/467	74.8 (2.33)‡	125/203	58.8 (4.38)	589/1001	61.4 (1.74)†	253/500	54.3 (2.60)
Emptiness	378/528	71.5 (2.39)	84/142	63.3 (4.55)	566/948	62.2 (2.05)‡	276/533	53.3 (2.49)
Dissociation/Paranoia	359/490	73.5 (2.29)‡	103/180	60.2 (4.18)	619/1040	62.5 (1.87)‡	223/461	51.4 (2.76)
Self-injurious Behavior	95/145	65.3 (4.85)	367/525	70.9 (2.33)	124/214	61.1 (3.94)	718/1287	58.7 (1.61)
Impulsivity	411/587	70.4 (2.18)	51/83	64.7 (7.01)	688/1232	59.5 (1.59)	154/269	56.7 (3.31)
**Any mood disorders**								
Unstable Relationship	480/563	87.1 (1.69)	93/107	88.3 (3.92)	397/886	71.1 (1.61)	156/218	70.5 (4.05)
Affective Instability	504/585	87.9 (1.49)	69/85	82.6 (6.07)	904/1290	72.1 (1.66)	138/211	63.9 (3.90)
Abandonment Fear	431/493	89.7 (1.46)‡	142/177	79.9 (3.83)	770/1124	69.9 (1.69)	272/377	74.1 (2.65)
Anger	448/531	85.9 (1.88)†	125/139	93.0 (2.17)	823/1191	70.4 (1.66)	219/310	73.6 (2.67)
Identity Disturbance	402/467	88.7 (1.61)	171/203	84.2 (3.31)	707/1001	72.5 (1.65)	335/500	68.0 (2.54)
Emptiness	462/528	88.2 (1.78)	111/142	83.4 (3.41)	704/948	76.6 (1.78)‡	338/553	61.2 (2.28)
Dissociation/Paranoia	424/490	88.4 (1.85)	149/180	84.4 (3.36)	752/1040	73.1 (1.69)†	290/461	66.4 (2.62)
Self-injurious Behavior	449/525	87.0 (1.78)	124/145	88.5 (2.96)	160/214	74.5 (3.34)	882/1287	70.5 (1.51)
Impulsivity	502/587	87.4 (1.72)	71/83	86.4 (3.79)	869/1232	72.2 (1.63)	173/269	65.7 (3.29)
**PTSD**								
Unstable Relationship	293/563	44.2 (2.41)	48/107	47.5 (5.87)	441/1283	32.8 (1.63)	69/218	27.4 (3.44)
Affective Instability	309/585	54.8 (2.76)‡	32/85	31.0 (6.23)	454/1290	33.3 (1.56)†	56/211	22.8 (3.53)
Abandonment Fear	264/493	54.6 (2.56)†	77/177	43.3 (5.04)	388/1124	33.1 (1.60)	122/377	28.7 (3.02)
Anger	268/531	51.0 (2.90)	73/139	55.0 (6.04)	410/1191	32.3 (1.71)	100/310	30.4 (2.88)

© 2021 Grilo CM et al. *JAMA Network Open*.

Identity Disturbance	256/467	56.9 (31.2)[†]	85/203	40.9 (5.00)	356/1001	33.5 (1.83)	154/500	28.6 (2.13)
s	With lifetime SA History				Without lifetime SA History			
	Met specific BPD criteria		Did not meet specific BPD criteria		Met specific BPD criteria		Did not meet specific BPD criteria	
	n/N	% (SE)	n/N	% (SE)	n/N	% (SE)	n/N	% (SE)
Emptiness	287/528	55.7 (2.76)[‡]	54/142	37.5 (4.65)	351/948	35.2 (1.84)[‡]	159/553	26.0 (2.19)
Dissociation/Paranoia	271/490	56.6 (30.5)[‡]	70/180	39.7 (3.97)	405/1040	37.3 (1.64)[‡]	105/461	20.0 (2.01)
Self-injurious Behavior	286/525	55.2 (2.85)[‡]	55/145	38.2 (5.33)	86/214	40.4 (4.54)[†]	424/1287	30.5 (1.49)
Impulsivity	306/587	52.7 (2.66)	35/83	45.5 (8.35)	432/1232	32.4 (1.54)	78/269	29.5 (2.93)
**Any substance use disorder**								
Unstable Relationship	484/563	87.0 (1.81)	82/107	83.6 (3.06)	968/1283	76.6 (1.32)	176/218	83.0 (2.95)
Affective Instability	493/585	85.6 (1.66)[‡]	73/85	92.7 (1.90)	974/1290	76.9 (1.29)	170/211	82.2 (3.34)
Abandonment Fear	427/493	87.9 (1.84)	139/177	82.1 (3.60)	865/1124	78.7 (1.40)	279/377	74.9 (2.48)
Anger	459/531	87.9 (1.90)	1074/139	80.7 (3.81)	928/1191	78.9 (1.38)[†]	216/310	72.8 (2.71)
Identity Disturbance	396/467	86.5 (1.80)	170/203	86.3 (3.08)	765/1001	79.1 (1.36)	379/500	74.8 (2.30)
Emptiness	448/528	86.6 (1.58)	118/142	86.0 (3.76)	709/948	77.0 (1.53)	435/553	78.9 (1.97)
Dissociation/Paranoia	406/490	84.4 (1.75)[†]	160/180	91.7 (2.29)	780/1040	75.9 (1.63)[†]	364/461	81.5 (1.97)
Self-injurious Behavior	448/525	86.6 (1.58)	118/145	85.7 (3.57)	174/214	81.7 (3.36)	970/1287	77.0 (1.30)
Impulsivity	511/587	88.8 (1.54)[‡]	55/83	69.4 (6.53)	994/1232	82.3 (1.34)[‡]	150/269	55.6 (3.63)
**Schizotypical personality disorder**								
Unstable Relationship	352/563	62.5 (2.88)[‡]	53/107	40.3 (4.69)	647/1283	50.4 (1.72)	112/218	51.6 (4.06)
Affective Instability	363/585	61.2 (2.75)[‡]	42/85	42.9 (6.61)	666/1290	51.8 (1.65)[†]	93/211	42.7 (4.05)
Abandonment Fear	316/493	32.8 (3.00)[‡]	89/177	47.4 (4.56)	571/1124	50.3 (1.73)	188/377	51.2 (3.45)
Anger	341/531	62.1 (2.87)[‡]	64/139	46.5 (5.40)	607/1191	51.3 (1.71)	152/310	47.7 (3.44)
Identity Disturbance	313/467	65.6 (2.93)[‡]	92/203	44.4 (4.90)	560/1001	55.4 (1.79)[‡]	199/500	40.8 (2.84)
Emptiness	342/528	63.0 (2.95)[‡]	63/142	44.0 (5.33)	528/948	56.2 (1.89)[‡]	231/553	40.5 (2.46)
Dissociation/Paranoia	338/490	69.2 (2.91)[‡]	67/180	32.9 (4.47)	593/1040	56.8 (1.74)	166/461	36.9 (2.91)
Self-injurious Behavior	316/525	58.8 (2.77)	89/145	59.4 (5.20)	125/214	58.8 (4.39)[†]	634/1287	49.3 (1.55)
Impulsivity	366/587	61.6 (2.70)[‡]	39/83	39.8 (7.83)	645/1232	52.0 (1.61)[†]	114/269	43.9 (3.67)
**Antisocial personality disorder**								
Unstable Relationship	180/563	35.2 (2.52)	23/107	23.9 (5.46)	229/1283	18.8 (1.67)	47/218	23.3 (3.55)

© 2021 Grilo CM et al. *JAMA Network Open*.

Affective Instability	185/585	34.3 (2.53)	18/85	26.8 (7.18)	241/1290	19.2 (1.66)	35/211	22.2 (3.42)
Abandonment Fear	163/493	35.6 (2.65)	40/177	26.6 (4.63)	210/1124	19.6 (1.74)	66/377	19.5 (2.67)
	With lifetime SA History				Without lifetime SA History			
	Met specific BPD criteria		Did not meet specific BPD criteria		Met specific BPD criteria		Did not meet specific BPD criteria	
	n/N	% (SE)	n/N	% (SE)	n/N	% (SE)	n/N	% (SE)
Anger	173/531	34.7 (2.70)	30/139	28.3 (4.51)	255/1191	22.8 (1.82)‡	21/310	6.6 (1.66)
Identity Disturbance	157/467	37.4 (2.71)‡	46/203	24.7 (3.79)	192/1001	20.8 (1.75)	84/500	17.0 (2.49)
Emptiness	164/528	34.6 (2.51)	39/142	29.1 (4.85)	170/948	19.9 (1.81)	106/553	18.9 (2.22)
Dissociation/Paranoia	154/490	35.0 (2.62)	49/180	29.4 (5.29)	184/1040	18.9 (1.80)	92/461	21.1 (2.92)
Self-injurious Behavior	156/525	34.7 (2.74)	47/145	28.4 (3.99)	38/214	17.9 (3.07)	238/1287	19.8 (1.63)
Impulsivity	196/587	36.7 (2.59)‡	7/83	9.2 (4.22)	250/1232	21.5 (1.76)‡	26/269	10.3 (1.83)
**Conduct disorder**								
Unstable Relationship	184/563	36.0 (2.48)	24/107	24.1 (5.46)	234/1283	19.4 (1.65)	41/218	25.4 (3.69)
Affective Instability	188/585	35.0 (2.52)	20/85	27.5 (7.16)	247/1290	20.0 (1.67)	36/211	22.4 (3.40)
Abandonment Fear	168/493	36.5 (2.63)	40/177	26.6 (4.63)	217/1124	20.6 (1.77)	66/377	19.5 (2.67)
Anger	176/531	35.4 (2.68)	32/139	28.7 (4.50)	261/1191	23.7 (1.86)‡	22/310	6.7 (1.58)
Identity Disturbance	161/467	38.3 (2.69)‡	47/203	24.8 (3.78)	198/1001	21.8 (1.75)	85/500	17.4 (2.50)
Emptiness	169/528	35.4 (2.54)	39/142	29.1 (4.85)	174/948	20.2 (1.80)	109/553	20.5 (2.22)
Dissociation/Paranoia	158/490	35.8 (2.58)	50/180	29.5 (5.30)	181/1040	19.1 (1.80)	97/461	23.1 (2.98)
Self-injurious Behavior	159/525	35.4 (2.76)	49/145	28.9 (4.01)	38/214	17.9 (3.07)	245/1287	20.7 (1.64)
Impulsivity	199/587	37.2 (2.57)‡	9/83	10.9 (4.37)	254/1232	21.9 (1.78)‡	29/269	12.8 (2.30)
**Childhood adverse experiences**								
Unstable Relationship	513/563	91.8 (1.26)	99/107	92.5 (3.22)	1088/1283	82.6 (1.15)	182/218	84.2 (2.84)
Affective Instability	538/585	92.8 (1.20)	74/85	85.4 (4.49)	1092/1290	82.5 (1.30)	178/211	85.5 (2.83)
Abandonment Fear	456/493	92.5 (1.41)	156/177	90.0 (2.62)	951/1124	82.6 (1.17)	319/377	83.6 (2.21)
Anger	491/531	93.2 (1.12)	121/139	86.5 (4.19)	1012/1191	83.3 (1.30)	258/310	81.1 (2.94)
Identity Disturbance	435/467	93.8 (1.15)†	177/203	87.6 (2.81)	851/1001	83.9 (1.51)	419/500	80.9 (2.00)
Emptiness	483/528	92.8 (1.06)	129/142	88.4 (3.06)	808/948	83.6 (1.46)	462/553	81.6 (2.20)
Dissociation/Paranoia	453/490	93.1 (1.11)	159/180	88.7 (2.47)	878/1040	81.8 (1.51)	392/461	85.3 (2.12)

© 2021 Grilo CM et al. *JAMA Network Open*.

Self-injurious Behavior	478/525	91.3 (1.34)	134/145	94.0 (2.40)	177/214	81.7 (3.41)	1093/1287	83.1 (1.28)
Impulsivity	541/587	92.9 (1.13)†	71/83	84.2 (5.46)	1056/1232	84.2 (1.24)†	214/269	76.9 (2.89)

Notes. All analyses were adjusted for the NESARC complex survey design. † = significantly different between respondents reporting a specific criterion and who did not at $p < .05$; ‡ = significantly different between respondents reporting a specific criterion and who did not at $p < .01$.

© 2021 Grilo CM et al. *JAMA Network Open*.
